# Ancient DNA Tells Story of Giant Eagle Evolution

**DOI:** 10.1371/journal.pbio.0030020

**Published:** 2005-01-04

The recent discovery of a Hobbit-like hominid on the Indonesian island of Flores was startling in some respects—its rather modern existence, for one—but it represents a classic case of Darwinian evolution. For reasons that are not entirely clear, when animals make their way to isolated islands, they tend to evolve relatively quickly toward an outsized or pint-sized version of their mainland counterpart. Following this evolutionary script, the Flores woman, presumably a downsized version of Homo erectus, appears to have shared her island home with dwarf elephants and giant rats.

Perhaps the most famous example of an island giant—and, sadly, of species extinction—is the dodo, once found on the Indian Ocean island of Mauritius. When the dodo's ancestor (thought to be a migratory pigeon) settled on this island with abundant food, no competition from terrestrial mammals, and no predators, it could survive without flying, and thus was freed from the energetic and size constraints of flight. New Zealand also had avian giants, now extinct, including the flightless moa, an ostrich-like bird, and Haast's eagle (Harpagornis moorei), which had a wingspan up to 3 meters. Though Haast's eagle could fly—and presumably used its wings to launch brutal attacks on the hapless moa—its body mass (10–14 kilograms) pushed the limits for self-propelled flight.

As extreme evolutionary examples, these island birds can offer insights into the forces and events shaping evolutionary change. In a new study, Michael Bunce et al. compared ancient mitochondrial DNA extracted from Haast's eagle bones with DNA sequences of 16 living eagle species to better characterize the evolutionary history of the extinct giant raptor. Their results suggest the extinct raptor underwent a rapid evolutionary transformation that belies its kinship to some of the world's smallest eagle species.[Fig pbio-0030020-g001]

**Figure pbio-0030020-g001:**
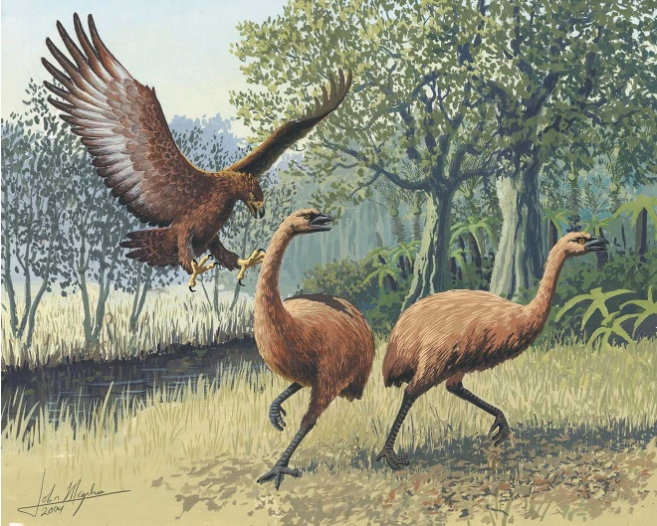
Giant Haast's eagle attacking New Zealand moa (Art: John Megahan)

The authors characterized the rates of sequence evolution within mitochondrial DNA to establish the evolutionary relationships between the different eagle species. Their analysis places Haast's eagle in the same evolutionary lineage as a group of small eagle species in the genus Hieraaetus. Surprisingly, the genetic distance separating the giant eagle and its more diminutive Hieraaetus cousins from their last common ancestor is relatively small.

Without the fossils to directly determine divergence times, Bunce et al. relied on molecular dating techniques that use the rate of sequence evolution in the genes studied to establish the relative evolutionary ages of the eagles. Proposing a divergence date of roughly 0.7–1.8 million years ago, the authors acknowledge that while this is the “best available approximation of the ‘true’ date,” additional molecular data could help refine the estimate. Whatever the date of divergence, the extinct giant eagle is clearly an anomaly among the eagles studied here. The increase in body size—by at least an order of magnitude in less than 2 million years—is particularly remarkable, Bunce et al. argue, since it occurred in a species still capable of flight.

The absence of mammalian competitors facilitated the evolution of much larger eagles and owls on Cuba and may have likewise precipitated the rapid morphological shift seen here. Haast's eagle, the authors write, “represents an extreme example of how freedom from competition on island ecosystems can rapidly influence morphological adaptation and speciation.” Given its similarity to the smaller Hieraaetus species, the authors recommend reclassifying the New Zealand giant as Hieraaetus moorei. This study shows how quickly morphological changes can occur in vertebrate lineages within island ecosystems. Could it be that anthropologists might some day uncover evidence of a giant version of the Flores woman?

